# Molecular Signaling Network Motifs Provide a Mechanistic Basis for Cellular Threshold Responses

**DOI:** 10.1289/ehp.1408244

**Published:** 2014-08-12

**Authors:** Qiang Zhang, Sudin Bhattacharya, Rory B. Conolly, Harvey J. Clewell, Norbert E. Kaminski, Melvin E. Andersen

**Affiliations:** 1 Institute for Chemical Safety Sciences, The Hamner Institutes for Health Sciences, Research Triangle Park, North Carolina, USA; 2 Integrated Systems Toxicology Division, National Health and Environmental Effects Research Laboratory, U.S. Environmental Protection Agency, Durham, North Carolina, USA; 3 Department of Pharmacology & Toxicology, and; 4 Center for Integrative Toxicology, Michigan State University, East Lansing, Michigan, USA

## Abstract

Background: Increasingly, there is a move toward using *in vitro* toxicity testing to assess human health risk due to chemical exposure. As with *in vivo* toxicity testing, an important question for *in vitro* results is whether there are thresholds for adverse cellular responses. Empirical evaluations may show consistency with thresholds, but the main evidence has to come from mechanistic considerations.

Objectives: Cellular response behaviors depend on the molecular pathway and circuitry in the cell and the manner in which chemicals perturb these circuits. Understanding circuit structures that are inherently capable of resisting small perturbations and producing threshold responses is an important step towards mechanistically interpreting *in vitro* testing data.

Methods: Here we have examined dose–response characteristics for several biochemical network motifs. These network motifs are basic building blocks of molecular circuits underpinning a variety of cellular functions, including adaptation, homeostasis, proliferation, differentiation, and apoptosis. For each motif, we present biological examples and models to illustrate how thresholds arise from specific network structures.

Discussion and Conclusion: Integral feedback, feedforward, and transcritical bifurcation motifs can generate thresholds. Other motifs (e.g., proportional feedback and ultrasensitivity)produce responses where the slope in the low-dose region is small and stays close to the baseline. Feedforward control may lead to nonmonotonic or hormetic responses. We conclude that network motifs provide a basis for understanding thresholds for cellular responses. Computational pathway modeling of these motifs and their combinations occurring in molecular signaling networks will be a key element in new risk assessment approaches based on *in vitro* cellular assays.

Citation: Zhang Q, Bhattacharya S, Conolly RB, Clewell HJ III, Kaminski NE, Andersen ME. 2014. Molecular signaling network motifs provide a mechanistic basis for cellular threshold responses. Environ Health Perspect 122:1261–1270; http://dx.doi.org/10.1289/ehp.1408244

## Introduction

Quantitative human health risk assessment for environmental toxicants requires accurate dose–response information on relevant end points. Dose–response studies using animals, tissues, and cells can examine diverse end points. However, scientists in the toxicology and risk assessment community are still at odds over the issue of whether threshold doses exist for adverse responses (Crawford and Wilson 1996; Crump et al. 1976; Piersma et al. 2011; Rhomberg et al. 2011). The debate on the existence of a threshold dose (a dose below which there is no increase in an adverse response, and above which the adverse response increases) has important implications for risk assessment. Without solid scientific backing for the shape of dose–response curves, particularly in the low-dose region, government agencies use linear non-threshold extrapolation models as default risk assessment tools [National Research Council (NRC) 2009].

Two issues are at the basis of the current challenges in providing convincing evidence for thresholds. First, it is difficult, if not impossible, to identify thresholds with certainty from experimental dose–response data alone. Although statistical tools can determine responses that are significantly different from the nonexposed control, lack of statistical significance between treated and control groups may arise from relatively small sample size and experimental variability rather than absence of actual change in response (Crump 2011; Lovell 2000). These statistical challenges are present when analyzing both *in vivo* and *in vitro* toxicity data (Crump et al. 2010). The second issue lies in our poor understanding of the mechanistic underpinnings of biological thresholds. One commonly voiced argument for thresholds is the capability of homeostasis to make a biological system resilient to small perturbations before breaking down (Piersma et al. 2011). There are also arguments for dose–response behaviors based on specific modes of action. For instance, genotoxic chemicals are thought to be low-dose linear because they add an increment of DNA damage over background; conversely, others in the toxicology community argue that nongenotoxic carcinogens have thresholds because they act through modes of action related to receptor activation or regenerative hyperplasia (U.S. Environmental Protection Agency 1986, 2005). Regarding endocrine-disrupting chemicals, some researchers have argued for thresholds based on the notion that biological systems have to be able to distinguish bona fide hormone signals from similarly structured background endogenous and exogenous molecules (Borgert et al. 2013). These broad arguments do not provide a compelling mechanistic understanding for threshold behaviors.

**Figure 1:**
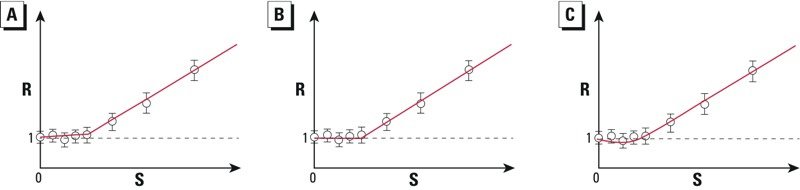
Schematics of dose–response curves at low chemical doses. Abbreviations: R, response; S, cellular stressor. (*A*) Dose–response curve with a small non-zero slope in the low-dose region before increasing significantly at higher doses. (*B*) Threshold dose–response curve that remains flat in the low-dose region and increases significantly once S exceeds the threshold. (*C*) Hormetic dose–response curve in which the slope first decreases and then increases in the low-dose region. The identical hypothetical data points (circles) are overlaid on all three panels.

Lutz and Lutz (2009) developed a statistical curve-fitting approach, which adopts a hockey-stick threshold model once a linear model is rejected. But as these authors pointed out, there is a great deal of uncertainty regarding the curvature in the low-dose region when one attempts to infer a threshold from dose–response data. No matter how strongly the dose–response data may appear to favor a threshold, the true curve can have several possible shapes that are quantitatively similar but qualitatively different (Figure 1). The curvature can be *a*) monotonically increasing but staying close to the control baseline (Figure 1A); *b*) superimposed on the baseline, representing a true threshold response (Figure 1B); or *c*) nonmonotonic [i.e., first decreasing then increasing (hormetic; Figure 1C)]. These curves are not easily distinguishable with routine curve-fitting algorithms (Lovell 2000). The statistically best-fit curve is not necessarily the true description of the response profile due to variability, sampling bias, and measurement errors.

Although empirical dose–response curves are essential for examining low-dose behaviors, optimally, evaluation of the evidence for thresholds also needs to take into account the primary biological mechanisms governing both control and treated responses (Edler and Kopp-Schneider 1998; Rhomberg et al. 2011). To do this we need to consider biological systems—cells, tissues, and organisms—as dynamic systems. The biology of the intrinsic control processes determines both the maintenance of the baseline-level responses and the dynamics of the responses to external perturbations. The molecular control networks operating in cells, organs, and whole organisms to maintain homeostasis are particularly important. Studies based on molecular and cellular systems biology have begun to map out details of the intracellular protein and gene networks mediating adverse responses of chemical perturbations (Caron et al. 2010; Oda and Kitano 2006). Physicists, mathematicians, and biomedical engineers have also made valuable contributions to the field of systems biology, bringing new perspectives to the quantitative understanding of molecular pathways and networks that control cellular responses to various stressors (Alon 2006; Tyson et al. 2003).

At the same time, new toxicity testing initiatives have arisen because of concerns for the humane use of animals and the failure of conventional animal-based toxicity studies to keep pace with evaluation of the large number of chemicals in commerce and new chemical products coming to market (NRC 2007; Safety Evaluation Ultimately Replacing Animal Testing 2012). These initiatives propose use of *in vitro* cellular systems based on perturbations of toxicity pathways. Toxicity pathways—preexisting protein and gene networks that, when sufficiently perturbed by chemicals, can lead to adverse health outcomes—can be examined in great detail with modern high-throughput molecular techniques. These toxicity pathways link molecular initiating events to modes of action and adverse outcome pathways (Ankley et al. 2010; Vinken 2013).

Here, we present an overview of basic molecular network structures in the context of threshold behaviors. These structures, known as “network motifs,” are fundamental building blocks of large, integrated signaling (toxicity) pathways. These motifs are more than theoretical concepts. They are ubiquitous in all biological signaling and control systems, and they are necessary for the integrity and normal functioning of cells in response to physiological signals and in the face of environmental fluctuations. These motifs form the basis of the circuits underpinning integrated cellular functions, including adaptation, homeostasis, proliferation, differentiation, and apoptosis (Alon 2007; Tyson and Novak 2010; Zhang et al. 2013a). In this review we illustrate the structures of these network motifs, examine the implications for dose responses in the low-dose region, and discuss the functional contexts in which these motifs operate.

## Network Motifs and Threshold Responses

Human intuition for thresholds comes primarily from our everyday experience with the surrounding physical world (e.g., the requirement for a certain amount of force to turn on a light switch). In contrast to mechanical thresholds, the manner in which thresholds arise for the molecular signaling pathways and networks that underlie cellular responses is less intuitive. According to mass action, the strength of initial interaction between a chemical and its direct molecular target is proportional to the free concentrations of the reactants. A suite of subsequent biochemical processes can transform these linear, molecular initiating events into responses that may or may not have thresholds.

Increasingly, biologists and biomedical engineers regard biological responses in terms of systems-level behaviors of dynamic networks. In this section, we discuss network motifs that generate dose–response profiles similar to those shown in Figure 1. We first describe common network motifs that underlie cellular adaptation and homeostasis; these motifs utilize negative feedback and incoherent feedforward loops. We then draw on the concept of bifurcations from dynamic systems theory to illustrate several threshold mechanisms, including saddle-node bifurcations, transcritical bifurcations, and supercritical pitchfork bifurcations. Finally, we use molecular titration to examine dose responses expected for certain “ultrasensitive” motifs. For each motif, we present an intuitive explanation for its dose–response behavior, give relevant biological examples, and discuss the functional context in which they operate. For more quantitatively oriented readers, mathematical models illustrating the underlying kinetics that govern the dose–response behaviors imparted by these motifs are provided in Supplemental Material, “SBML XML Files.”

## Homeostatic Network Motifs

One of the most vital properties of biological organisms is homeostasis (i.e., the ability to resist or adapt to moderate levels of external perturbations and maintain a relatively stable internal environment). Homeostasis takes place over various levels of biological hierarchy: in cells, tissues, organs, and the whole organism. Adaptation and homeostasis are the mechanisms most frequently cited in the literature in support of thresholds in biological organisms (Piersma et al. 2011). Below we illustrate how two common network motifs—negative feedback and incoherent feedforward—produce adaptation and homeostasis, as well as response thresholds.

**Figure 2:**
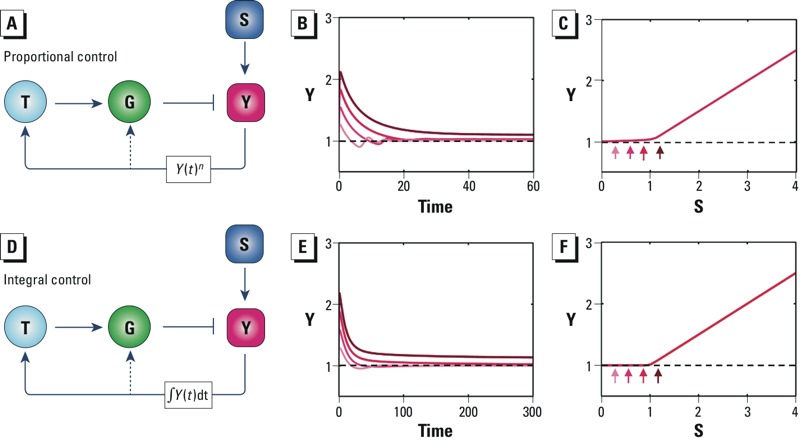
Negative feedback motifs. Abbreviations: G, stress genes; S, cellular stressor; T, transcription factor; Y, controlled variable. *Y*(*t*)*^n^* denotes mathematically ultrasensitive activation of T and G by Y; ∫*Y*(*t*)dt denotes mathematically time-integral activation of T and G by Y. (*A*) Schematic of a proportional feedback control motif to counter cellular stress. (*B*) With high-loop signal amplification, proportional feedback produces near-perfect adaptation, where Y settles asymptotically close to the baseline (i.e., the dashed line for S = 0). (*C*) Proportional feedback produces steady-state dose–response curves where the low-dose region is very close to the baseline. (*D*) Schematic of an integral feedback control motif to counter cellular stress. (*E*) Within stressor limits, integral feedback produces perfect adaptation (i.e., Y settles back exactly to the baseline). (*F*) Integral feedback produces well-defined thresholds for the steady-state response where the low-dose region overlaps with the baseline. In *A* and *D*, solid arrow heads denote activation and blunted arrow heads denote inhibition. In *B* and *E*, increasingly darker red lines correspond to S levels of 0.3, 0.6, 0.9, and 1.2. In *C* and *F*, small arrows indicate steady-state responses of Y associated with S levels of 0.3, 0.6, 0.9, and 1.2.

*Negative feedback control.* In negative feedback, an undesirable change brought about by a stressor functions as a signal that works to restore the system to its original condition. Negative feedback loops are frequently encountered in molecular circuits underlying signal attenuation, metabolic control, and most relevant to toxicologists, maintenance of cellular homeostasis (Cirit et al. 2010; Novak and Tyson 2008; Zhang et al. 2010c). For the latter function, the negative feedback mechanism has been primarily studied in relation to gene regulatory networks activated by cellular stresses (Zhang and Andersen 2007). Such networks (Figure 2A,D) typically contain a master transcription factor (T), with an associated sensor molecule that detects changes in certain specific cellular states (controlled variables; Y). These cellular states could be levels of reactive oxygen species, oxygen molecules (O_2_), DNA damage, protein folding, metal ions, or osmolarity (Simmons et al. 2009). Once activated, the transcription factor induces a suite of stress genes (collectively represented by G) to counteract changes in the controlled variable brought about by the cellular stressor (S). In certain cases, the controlled variable directly regulates the activities of the stress gene products (usually specific enzymes) in a posttranslational manner, forming a short feedback loop that bypasses the transcriptional control (Figure 2A,D, dashed lines). Depending on how the transcription factor and stress genes respond to changes in the controlled variable, the feedback control can be either “proportional” or “integral.”

Proportional feedback control. Proportional feedback is a term borrowed from engineering and implies that the output of the controller (e.g., the activity of T or G) is proportional to the error signal (e.g., the amount of deviation of Y from the baseline level). In a biological setting, such a definition can be sometimes confusing because Y may activate T or G in a nonlinear manner (Zhang and Andersen 2007). Regardless, an important characteristic of this type of feedback control is that the activities of T and G are related to the current value of Y rather than the past or projected future. Conceivably, the error signal of Y can be processed through certain signal amplification mechanisms that drive a strong induction of the stress gene G, causing the stress-induced departure of Y from the baseline to be minimized. Indeed, stress signaling pathways often embed ultrasensitive response motifs, which are key network motifs that amplify biochemical signals (Goldbeter and Koshland 1982, 1984) (for more information on ultrasensitivity, see “Ultrasensitive network motifs” below). With strong amplification in the negative feedback loop, culminating in high stress–gene induction, the perturbation can be nearly completely counteracted. In the continued presence of the stressor, the time-course response of the controlled variable would gradually return to the baseline; however, it would not settle to a steady state that is exactly equal to the original value (Figure 2B). The steady-state dose–response curve in the low-dose region can remain close to the baseline with a slope much smaller than in the absence of feedback (Figure 2C). As the stressor level increases further, causing stress-gene induction to reach its limit (such as due to maximal promoter occupancy by the transcription factor), the response profile of the controlled variable rises much more steeply (Figure 2C). A mathematical model illustrating the dose response expected for proportional feedback control is provided in Supplemental Material, “Proportional Feedback Control.”

Many cellular stress-response pathways contain negative feedback loops with multiple ultrasensitive motifs embedded along the feedback loops to enhance signal amplification (Zhang and Andersen 2007). With the oxidative stress response, activation of the master transcription factor Nrf2 (nuclear factor E2-related factor 2) by reactive oxygen species occurs through multistep signaling and by transcriptional autoregulation, both of which are ultrasensitive in nature (Zhang et al. 2010c, 2013a). Many antioxidant enzymes form homodimers or homotetramers after transcriptional induction, further increasing signal amplification along the feedback loop (Zhang et al. 2010c). With the hypoxic response, ultrasensitive activation of transcription factor HIF-1 (hypoxia-inducible factor-1) by low O_2_ occurs through multistep signaling (protein stabilization and transactivation) and molecular titration (Schmierer et al. 2010; Semenza 2004). For the heat shock response, ultrasensitive motifs increasing loop amplification include homotrimerization of transcription factor HSF1 (heat shock factor protein 1) and cooperative binding of the HSF1 trimer to the promoters of heat shock genes (Liu and Thiele 1999; Xiao et al. 1991). Most of these stress-response pathways also involve activation of MAPK cascades, a set of well-characterized ultrasensitive signaling motifs (Huang and Ferrell 1996). These ultrasensitive processes function collectively to provide a high degree of amplification for proportional feedback control. With high amplification, these pathways can produce muted steady-state responses between the stressor and the perturbed cellular state (Figure 2C) (i.e., the incremental increase above background in the low-dose region is much smaller compared to that in the absence of feedback control).

Integral feedback control. Integral feedback control is one of the few network topologies that can achieve perfect adaptation, where the perturbed state returns exactly to the preperturbation state even in the continued presence of the stressor (Ma et al. 2009). In contrast to proportional feedback control, integral control requires that the output of the controller be related to the time integral of the error signal (i.e., what feeds into the controller is the cumulative past history of the error signal rather than the present state of the controlled variable). In the context of the stress signaling circuit (Figure 2D), integral feedback control occurs when the activity or abundance of T or G depends on the time integral of the difference of Y from the baseline (ΔY). This integral over time is equivalent to the area under the curve (AUC), where the *x*-axis is time and the *y*-axis is ΔY. When the stressor increases causing the controlled variable Y to rise above the baseline, the AUC increases leading to induction of stress gene G, which in turn brings Y down. As long as Y does not return to the baseline completely, the AUC will continue to increase, causing more induction of G, further reducing Y. This adaptive process goes on until Y returns exactly to the baseline where the AUC and hence G stay constant (Figure 2E). In this fashion, the integral feedback control motif achieves perfect adaptation. This perfect adaptation breaks down when the system reaches the point of maximum induction of G, where it exhibits a threshold for Y (Figure 2F). A model illustrating how zero-order protein degradation makes a molecular integrator and how integral control produces perfect adaptation and well-defined thresholds is provided in Supplemental Material, “Integral Feedback Control.”

Several biological homeostatic systems appear to operate by integral feedback control. A well-studied example is the yeast osmotic stress-response pathway (Muzzey et al. 2009). Yeast cells use the membrane proteins Sln1 and Sho1 to sense changes in cell volume and/or membrane geometry caused by osmotic shock. An intracellular signal transduction cascade, culminating in the activation of Hog1, a yeast homolog of MAPK, conveys stress signal downstream, initiating both posttranslational and transcriptional regulatory events (Mettetal et al. 2008). For low-level hyperosmotic stress, a rapid response occurs with Hog1 phosphorylation of glycerol-synthesizing enzymes such as Gpd1 in the cytosol and of glycerol transporter Fps1 on the cell membrane. Phosphorylation activates Gpd1 and inhibits Fps1, leading to rapid accumulation of intracellular glycerol to counteract the hyperosmotic stress. In a second pathway, high-level hyperosmotic stress causes Hog1 to translocate to the nucleus where it phosphorylates transcription factor Hot1 and activates an antistress transcriptional program. Regardless of the pathways activated, a molecular integrator for integral control is located downstream of Hog1, underpinning the perfect adaptation to osmotic shock in yeast (Muzzey et al. 2009). Other cellular systems that appear to utilize integral feedback control to achieve perfect regulation are bacterial ammonium homeostasis (Kim et al. 2012) and chemotaxis (Yi et al. 2000). At higher biological organization levels, integral control underpins organ pattern formation through mechanical or paracrine feedback among proliferating cells (Lander 2011; Shraiman 2005), and mammalian calcium and glucose homeostasis through hormonal regulation (El-Samad et al. 2002; Koeslag et al. 1997; Saunders et al. 1998). The design principles of integral feedback control for natural and synthetic biological systems, in consideration of saturable enzymatic removal of integrator protein, diluting effect of cell growth, and the scope of control processes, have been widely studied (Ang and McMillen 2013; Ang et al. 2010; Ni et al. 2009).

**Figure 3:**
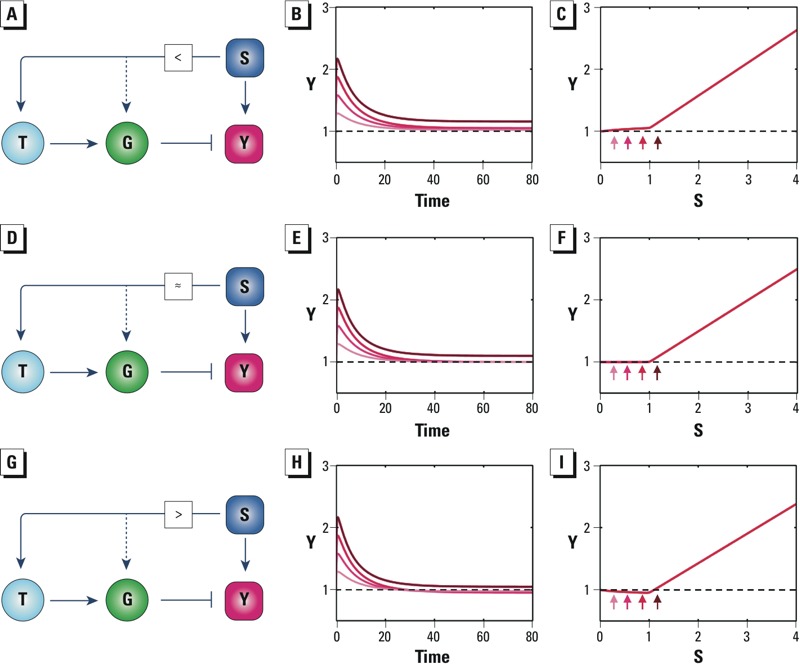
Incoherent feedforward motifs. Abbreviations: G, stress genes; S, cellular stressor; T, transcription factor; Y, controlled variable. (*A*) Schematic of an incoherent feedforward motif with feedforward signaling (S→T→GY) strength that is smaller than the perturbation (S→Y) strength. (*B*–*C*) Smaller feedforward signaling gain leads to partial adaptation and greatly limits the steady-state increases in Y in the low-dose region. (*D*) Schematic of an incoherent feedforward motif with feedforward signaling strength equal to perturbation strength. (*E*–*F*) Matching feedforward signaling strength leads to perfect adaptation and well-defined thresholds for the steady-state response. (*G*) Schematic of an incoherent feedforward motif with feedforward signaling strength greater than perturbation strength. (*H*–*I*) Greater feedforward signaling strength leads to overadaptation and hormetic steady-state response. In *B*, *E*, and *H*, increasingly darker red lines correspond to S levels of 0.3, 0.6, 0.9, and 1.2. In *C*, *F*, and *I*, small arrows indicate steady-state responses of Y associated with S levels of 0.3, 0.6, 0.9, and 1.2.

*Incoherent feedforward control.* Another network motif that can underpin adaptation and threshold response is incoherent feedforward control (Ma et al. 2009). Here we have a control mechanism where the stressor itself and the responses activated by the stressor work in concert to mitigate the cellular changes caused by the stressor. With this motif, there are sensing mechanisms that detect the presence of the stressor itself rather than the deviation of the controlled variable from a set point (Figure 3). Here, the feedforward path, S→T→GY, induces the stress gene G to compensate for changes in Y brought about by S. As with feedback control, posttranslational activation of the protein product of gene G by S may also occur (Figure 3A,D,G; dashed lines). Depending on the signaling strength of the feedforward path, perturbation of Y can be undercompensated, perfectly compensated, or overcompensated, leading to partial (Figure 3B), perfect (Figure 3E), or overadaptation (Figure 3H), respectively. Correspondingly, the low-dose region of the steady-state dose–response curve may be monotonically increasing with a small slope (Figure 3C), superimposed on the baseline (Figure 3F), or nonmonotonic (i.e., slightly decreasing then increasing) (Figure 3I). The inflection point, equivalent to a threshold, occurs approximately at a stressor level where activation of T or G reaches their maximal induction. A model illustrating how varying feedforward signaling strength produces different degree of adaptation and different shapes of dose–response curves in the low-dose region is provided in Supplemental Material “Incoherent feedforward control.”

Because feedforward control responds to the stressor rather than the state perturbed by the stressor, this type of control can be preemptive, capable of responding more quickly to the increased level of the stressor. Biological systems make frequent use of feedforward control, often in combination with negative feedback control. An example for cellular stress response is heat shock in *Escherichia coli*, where the thermosensor is the mRNA molecule of transcriptional factor σ^32^ (Morita et al. 1999). At normal temperatures, a special hairpin structure at the 5´-end of the mRNA molecule blocks efficient translation. At higher temperatures, the hairpin loop opens, allowing more efficient protein translation. The subsequent accumulation of σ^32^ protein induces gene expression of heat shock proteins and chaperons to rescue misfolded proteins accumulated due to heat shock. In combination with negative feedback control, this feedforward control mechanism allows faster and more robust adaptation to heat stress (El-Samad et al. 2005). Xenobiotic-metabolizing enzyme systems also appear to utilize feedforward control (Zhang et al. 2009). In the canonical phase I, II, and III chemical detoxification pathways activated by xenobiotics, phase I metabolic enzymes activate parent chemicals to produce reactive metabolites. These reactive metabolites can then induce phase II enzymes for their detoxification through a negative feedback loop. Importantly, in a feedforward manner, parent xenobiotic chemicals may also activate xenosensors such as AhR (aryl hydrocarbon receptor), CAR (constitutive androstane receptor), and PXR (pregnane X receptor), which directly induce phase II enzymes to detoxify reactive metabolites formed by phase I oxidative reactions. These feedforward control schemes (i.e., phase I to phase II cross-induction of xenobiotic-metabolizing enzymes) may provide a basis for threshold or hormetic effects observed with some xenobiotic chemicals (Zhang et al. 2009). Cells also use transcriptional incoherent feedforward circuits, such as those utilizing microRNAs as negative regulators, to achieve adaptation to changes in gene dosage (Bleris et al. 2011; Tsang et al. 2007). Recently, Takeda et al. (2012), studying the signal transduction pathway of eukaryotic chemotaxis, identified incoherent feedforward as the likely mechanism for perfect adaptation of Ras protein to activation by chemoattractant cAMP. Because incoherent feedforward control is not a robust adaptation mechanism by itself, it often operates in concert with feedback processes, as in heat shock response and body temperature regulation (El-Samad et al. 2005; Houk 1988). These integrated systems allow fast, preemptive, and robust adaptation and homeostasis against various environmental perturbations.

## Bifurcation Network Motifs

Biological networks are dynamic systems with multiple interacting components (Tyson et al. 2003; Zhang et al. 2010a). A biochemical network may have multiple stable steady states representing different cellular outcomes. For example, a precursor cell may change to a differentiated cell type after receiving a transient signal in which the underlying genetic network moves from one stable steady state representing the precursor cell type to another stable steady state representing the differentiated cell type. Dynamic networks may change their stability behaviors qualitatively over very small changes in the strength of external perturbations (e.g., chemical concentration). In nonlinear dynamic theory this process is called a “bifurcation” (Strogatz 1994). These “bifurcation points” would be equivalent to thresholds in biological systems. Here we discuss three network motifs that generate biologically relevant bifurcations.

**Figure 4:**
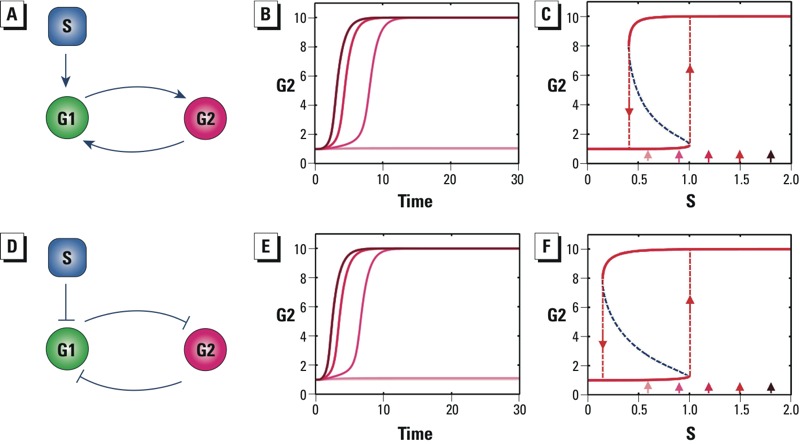
Saddle-node bifurcation motifs. Abbreviations: G1 and G2, gene 1 and gene 2; S, perturbing signal. (*A*) Schematic of a two-gene positive feedback system in which S activates G1. (*B*) Depending on the level of S, expression of gene G2 settles to either high or low levels. (*C*) The saddle-node bifurcation of G2 shows the steady-state dose response of G2 to S. (*D*) Schematic of a two-gene double-negative feedback system in which S inhibits G1. (*E*) Depending on the level of S, expression of G2 settles to either high or low levels. (*F*) The saddle-node bifurcation of G2 shows the steady-state dose response of G2 to S. In *B* and *E*, increasingly darker red lines correspond to S levels of 0.6, 0.9, 1.2, 1.5, and 1.8, where lines for S = 0.6 and S = 0.9 overlap. In *C* and *F*, small arrows indicate steady-state responses of G2 associated with S levels of 0.6, 0.9, 1.2, 1.5, and 1.8; dashed red lines with arrows define the on- and off-thresholds that delimit the bistable zone, and dashed blue lines denote unstable steady states.

*Saddle-node bifurcation.* In many nonlinear signaling systems, the signaling elements can give rise to discrete steady states. A saddle-node bifurcation is made by a network motif that produces abrupt dose-dependent transition in a system that can exist in one of two or more stable steady states (i.e., the system is capable of bistable or multistable switching). Bistable switching allows cells to undergo discontinuous, sometimes irreversible, changes in phenotypic state. Such switches drive integrated cellular functions such as cell cycle progression, metabolic switching, lineage specification, and differentiation (Ozbudak et al. 2004; Verdugo et al. 2013; Xiong and Ferrell 2003). The network motif structure of a bistable switch (Figure 4A,D) consists of a positive or double-negative feedback loop (Ferrell 2002). Here G1 and G2 are two genes/proteins mutually activating or inhibiting each other, and S is an external agent perturbing G1. With enough signal amplification (ultrasensitivity) between G1 and G2, the self-reinforcing nature of a positive or double-negative feedback loop allows the system to have two possible cellular states, either fully activated or not activated at all. When S is small, the feedback strength is weak; so G2 remains at low levels. When S is sufficiently large, the feedback increases in strength until it becomes self-sustaining, with G2 switching to a high level of expression (Figure 4B,E). This behavior allows perturbations below a threshold to be filtered out and suprathreshold perturbations to trigger switching to a new stable steady state with dramatically altered expression levels of G1 and G2. The steady-state dose–response behavior of a bistable switch is a saddle-node bifurcation that captures abrupt, discontinuous transitions (Figure 4C,F). The thresholds turning the cellular switch on and off are not equal, creating between them a zone where the system can be either stably on or off. This phenomenon—where at a given external condition, a system can reside in one of two available stable steady states—is known as bistability. A more quantitative explanation of the generation of thresholds from saddle-node bifurcations is provided in Supplemental Material, “Saddle-node bifurcation.”

The all-or-none nature of a bistable response makes it useful in many cellular processes that require binary decisions. Multiple bistable switches composed of positive and double-negative feedback loops control progression through various phases of the cell cycle (Verdugo et al. 2013). With genotoxic chemicals, which produce DNA damage and cause cell cycle arrest, sufficiently large amounts of damage appear to act on various cell cycle checkpoints to block the underlying bistable switches for cell cycle progression (Tyson et al. 2002). Through computational modeling we found that a gene network of coupled double-negative feedback loops is likely to be the basis of a bistable switch regulating terminal differentiation of mature B lymphocytes into antibody-secreting plasma cells (Bhattacharya et al. 2010). The environmental contaminant TCDD (2,3,7,8-tetrachlorodibenzo-*p*-dioxin) appears to disrupt the bistable switch triggered by antigens, leading to all-or-none suppression of B-cell differentiation (Zhang et al. 2013b). By perturbing naturally existing bistable switches, many environmental chemicals are likely to produce threshold responses through saddle-node bifurcations.

**Figure 5:**
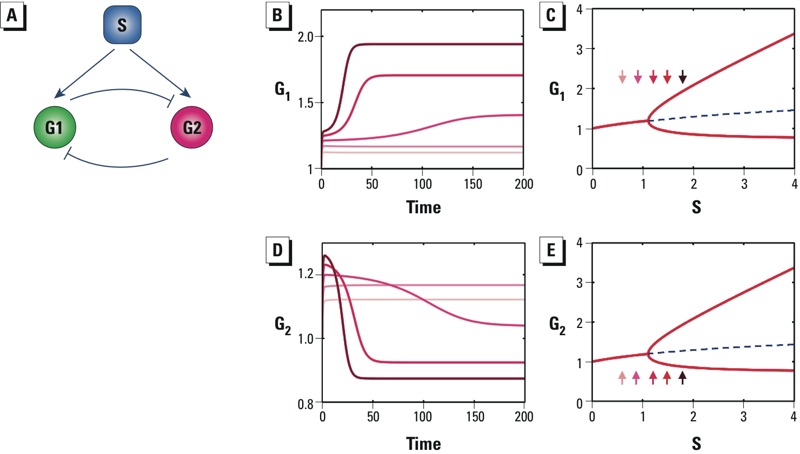
Supercritical pitchfork bifurcation motif. Abbreviations: G1 and G2, gene 1 and gene 2; S, perturbing signal. (*A*) Schematic of a symmetrical two-gene system in which genes G1 and G2 mutually repress each other and S equally activates both G1 and G2. (*B*) Dynamic (i.e., time-dependent) response of G1 to various levels of S. (*C*) Supercritical pitchfork bifurcation of G1 exhibits an abrupt transition in the steady-state dose response. (*D*) Dynamic response of G2 to various levels of S. (*E*) Supercritical pitchfork bifurcation of G2. In *B* and *D*, the initial value of G1 is set at 1.01 and the initial value of G2 is set at 1.0 to introduce slight asymmetry; increasingly darker red lines correspond to S levels of 0.6, 0.9, 1.2, 1.5, and 1.8. In *C* and *E*, small arrows indicate steady-state responses of G1 and G2 associated with S levels of 0.6, 0.9, 1.2, 1.5, and 1.8; dashed blue lines denote unstable steady states.

*Pitchfork bifurcation.* In a pitchfork bifurcation, increasing levels of perturbation cause the motif to move from an intermediate-level stable steady state to either a low- or a high-level stable steady state. It likely underlies lineage specification where a bipotent progenitor cell, in response to a differentiating signal, makes a binary decision to move to one or another sublineage (Huang et al. 2007). Balanced double-negative feedback loops can generate pitchfork bifurcations (Ferrell 2012; Widder et al. 2007). Unlike the double-negative feedback loop illustrated for saddle-node bifurcation (Figure 4D) in which the external signal impinges on one gene, this motif has a common signal S that equally affects both genes (Figure 5A). At low levels of perturbation by S, G1 and G2 rise equally. This balanced state represents a progenitor cell stage. As S drives G1 and G2 to further higher levels, the strength of mutual antagonism between G1 and G2 also increases, creating two new stable steady states: either high G1/low G2 or high G2/low G1. Any transient asymmetry between the rates of change of G1 and G2 will tilt the intermediate balanced state and move the cell to one of the two new stable steady states (Figure 5B,D). In the context of cell differentiation, the movement to one or the other stable steady state signals a choice of lineage specification. This type of phase transition is a supercritical pitchfork bifurcation: The system first monotonically increases and then bifurcates into a bistable phase characterized by opposing G1 and G2 levels (Figure 5C,E). A mathematical model of pitchfork bifurcation is provided in Supplemental Material, “Pitchfork bifurcation.”

With a balanced network structure, the subthreshold region of a pitchfork bifurcation can be a flat line with no changes. This would require a third gene, G3, which is activated by G1 and repressed by G2. The two opposing forces upon G3 exactly cancel each other out in regions where G1 and G2 are expressed at the same levels. Because subthreshold levels of S lead to equal expression G1 and G2, G3 remains unchanged from its basal level. At the bifurcation point where G1 and G2 move in opposite directions, G3 would either increase or decrease, producing a pitchfork bifurcation of its own. Its expression profile would be a threshold response with zero change in the subthreshold region.

Building upon the double-negative feedback loop, a more involved gene network motif arises when both genes, G1 and G2, positively autoregulate their own expression, forming two additional positive feedback loops. This motif is likely more common than a single double-negative feedback loop in the context of lineage commitment of bipotent progenitor cells (Huang et al. 2007). This modification of the motif structure can create a subcritical pitchfork bifurcation with three stable steady states (Foster et al. 2009; Guantes and Poyatos 2008). In theoretical models of common myeloid precursor cells choosing between either the erythroid or myelomonocytic fate, gene circuits involving GATA1 and PU.1 as mutually repressing transcription factors with positive autoregulation exhibit a subcritical pitchfork bifurcation (Huang et al. 2007). A similar pitchfork bifurcation was proposed for gene networks containing mutually repressing transcriptional repressors Foxp3 (forkhead box P3) and RORγt (RAR-related orphan receptor gamma 2). These two proteins underpin differentiation of naive CD4^+^ T cells into T helper 17 cells or induced regulator T cells (Hong et al. 2011). Environmental chemicals that target cell differentiating signals (e.g., S in Figure 5A) could potentially generate pitchfork bifurcations, thereby producing thresholds for developmental responses to chemicals.

**Figure 6:**
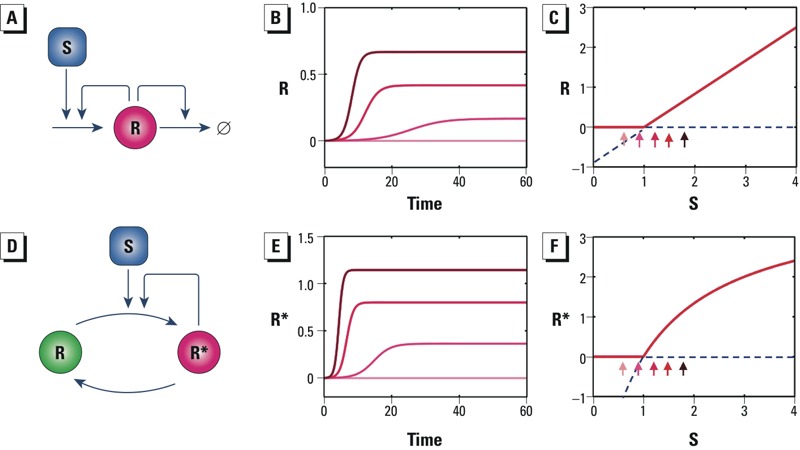
Transcritical bifurcation motifs. Abbreviations: ∅, degradation; R, protein; R*, covalently modified protein; S, perturbing signal. (*A*) Schematic of a motif where protein R promotes both its own synthesis and degradation. (*B*) Dynamic response of R to various levels of S. (*C*) Transcritical bifurcation of R produces a perfect steady-state threshold response. (*D*) Schematic illustration of an autocatalysis motif in which R is autocatalyzed to R*. (*E*) Dynamic response of R* to various levels of S. (*F*) Transcritical bifurcation of R* produces a perfect steady-state threshold response. In *B* and *E*, increasingly darker red lines correspond to S levels of 0.6, 0.9, 1.2, 1.5, and 1.8, where lines for S = 0.6 and S = 0.9 overlap. In *C* and *F*, small arrows indicate steady-state responses of R and R*, respectively, associated with S levels of 0.6, 0.9, 1.2, 1.5, and 1.8, and dashed blue lines denote unstable steady states (note the exchange of stability at the intersections).

*Transcritical bifurcation.* A transcritical bifurcation is the state transition of a dynamic system where a monostable steady state and an unstable steady state coexist and exchange their stability as the level of perturbation increases. As the strength of the stimulus increases, the two states move closer to each other, coalesce, and then exchange their stability after crossing (Strogatz 1994). Network motif structures underlying transcritical bifurcation include positive feedback regulation of protein production coupled with nonlinear clearance. In Figure 6A, protein R activates both its own synthesis and degradation. The synthesis rate of R is linearly related to its own concentration, and the degradation rate of R is a second-order function of R. At low levels of S, the synthesis rate of R is less than its degradation rate, so R always settles to zero as the stable steady state. Once S exceeds a critical level, the synthesis rate of R matches the degradation rate, creating positive-valued stable steady states (Figure 6B). A transcritical bifurcation produces a dose response with a threshold (Figure 6C). In a similar manner, autocatalysis with reversible reactions also generates transcritical bifurcation and thresholds (Figure 6D–F). Mathematical models of both of these motifs are provided in Supplemental Material, “Transcritical bifurcation.”

Model-based studies indicate that positive feedback loop motifs with mutually activating genes, protein covalent modification cycles with autocatalysis, and enzymatic cascades produce transcritical bifurcations (Aguda 1999; Alam-Nazki and Krishnan 2012; Widder et al. 2007). Positive and negative feedback regulation formed between transcription factor E2F and inhibitor protein RB may underlie a transcritical bifurcation for the restriction point transition from G_0_ to G_1_ in the cell cycle stimulated by mitogens (Swat et al. 2004). There is also support for transcritical bifurcation motifs controlling other cellular processes: *a*) activation of the extrinsic apoptosis pathway by tumor necrosis factor (Albeck et al. 2008); *b*) the phase transition between proliferation and extinction of RNA virus in response to variations in RNA proliferation mode and RNA strand degradation (Sardanyés et al. 2012); *c*) liver damage induced by HIV infection (Nampala et al. 2013); *d*) switching of excitability of neurons expressing both restorative and regenerative ion channels (Franci et al. 2013); and *e*) the transition from the quiescent to persistent firing states in neuronal networks when the network connectivity exceeds a threshold level (Droste et al. 2013). Environmental chemicals perturbing a toxicity pathway through a transcritical bifurcation would produce threshold dose–response behaviors.

## Ultrasensitive Network Motifs

**Figure 7:**
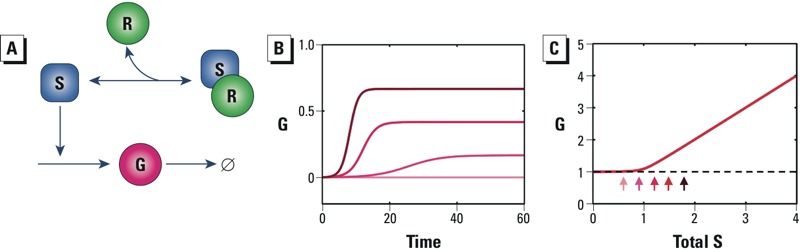
Example of an ultrasensitive motif. Abbreviations: ∅, degradation; G, gene G; R, stoichiometric inhibitor; S, stimulating signal. (*A*) Schematic of a molecular titration motif where inhibitor R sequesters S, reducing the ability of free S to induce gene G. (*B*) Dynamic response of G to various levels of total S. Increasingly darker red lines correspond to total S levels of 0.6, 0.9, 1.2, 1.5, and 1.8, where lines for S = 0.6 and S = 0.9 overlap. (*C*) Although not a perfect threshold response, the steady-state response of G to the total S level increases abruptly as total S increases above R. Small arrows indicate steady-state responses of G associated with S levels of 0.6, 0.9, 1.2, 1.5, and 1.8; the dashed line denotes the baseline.

Ultrasensitivity is a common type of nonlinear signal processing in molecular signaling networks where a small fractional change in the input generates a much larger fractional change in the output. These ultrasensitive motifs often produce sigmoidal dose–response curves (Goldbeter and Koshland 1982, 1984). A number of network motifs can generate ultrasensitive responses, including positive cooperative binding, homomultimerization, multistep signaling, molecular titration, zero-order covalent modification cycle, and positive feedback (Zhang et al. 2013a). Although the outputs of most ultrasensitive motifs are sigmoid, some motifs, including multisite phosphorylation (a form of multistep signaling) and molecular titration, may give rise to low-dose regions that would approximate a threshold response (Buchler and Cross 2009; Gunawardena 2005). With molecular titration (Figure 7A), there is a suppressed response in the low-dose region, which occurs because, in order to significantly activate gene G, the total amount of stimulus S has to exceed the total amount of the high-affinity inhibitor R that avidly sequesters S, preventing activation of G (Figure 7B,C). A model of molecular titration producing near-threshold dose response is provided in Supplemental Material, “Molecular titration.”

Molecular titration and multisite phosphorylation/dephosphorylation are very common ultrasensitive network motifs. In the hypoxic stress pathway, factor inhibiting HIF (FIH) hydroxylates HIF-1α but also has a range of ankyrin-repeat domain (ARD)-containing proteins as substrate (Cockman et al. 2009). The molecular titration of FIH by ARD-containing proteins likely determines the threshold (i.e., extent of decreased oxygen tension for activation of HIF-1α) (Schmierer et al. 2010). Small RNAs, which titrate target mRNAs, may also be responsible for some threshold responses in gene and protein expression (Legewie et al. 2008; Lenz et al. 2004; Levine et al. 2007; Mehta et al. 2008; Mukherji et al. 2011). Synthetic biology studies that engineered high-affinity molecular inhibitors into cellular systems provide clear experimental evidence for ultrasensitive threshold response via molecular titration (Buchler and Cross 2009; Lee and Maheshri 2012). With respect to multisite phosphorylation or dephosphorylation, pathway simulation predicted that activation of transcription factor NFAT1 (nuclear factor of activated T cells) is ultrasensitive with a threshold (Salazar and Hofer 2003). This pathway requires dephosphorylation of 13 serine residues by calmodulin-dependent phosphatase calcineurin to activate NFAT1 (Okamura et al. 2000). Multisite phosphorylation of Cdc25C (cell division cycle 25C) by Cdk1 (cyclin-dependent kinase 1), two key components involved in cell cycle regulation in *Xenopus* oocytes, also exhibited an ultrasensitive response (Trunnell et al. 2011).

## Discussion

Dose–response relationships are at the core of quantitative toxicology and chemical risk assessment. Determining the shape of dose–response curves in the low-dose region to assess evidence for thresholds based on statistical analyses has proven difficult experimentally and unattainable theoretically. Due to biological and experimental variability, a statistical threshold does not necessarily indicate the existence of a real biological threshold, nor does a real biological threshold necessarily lead to an observable statistical threshold. Mechanistic knowledge of relevant biological networks and toxicity pathways perturbed by chemicals of health concern, as we show here, will be increasingly important in providing the biological underpinning for threshold responses.

Threshold effects can arise from simple network structures, commonly referred to as network motifs. Here, we have reviewed a number of network motifs that can underlie biological threshold responses at the cellular level. We have also described simple mathematical models for the motifs and examined their dose–response behaviors (Figures 2–7; see also Supplemental Material). In addition, we evaluated experimental work and combined experimental/theoretical work that provided evidence for these motifs in specific biological processes. Among the motifs described for homeostasis, integral feedback and incoherent feedforward with matching feedforward and perturbation strengths produce perfect adaptation with clear-cut, mechanistically definable thresholds. With these motifs, there is a particular dose (threshold) below which steady-state responses are identical to those in the nonstressed controls. Some bifurcation network motifs also have well-defined thresholds; however, the expected curve shapes in the region near the threshold dose may be diverse. Transcritical bifurcations and supercritical pitchfork bifurcations can have flat subthreshold behaviors similar to that of integral feedback, and their shapes above the threshold dose increase gradually. In contrast, saddle-node bifurcations produce a discontinuous change in response at a particular perturbation point. This kind of dose response is reminiscent of true switch-like behaviors controlling key cellular phenotype changes.

Some network motifs, including proportional feedback loops, incoherent feedforward loops, and ultrasensitive motifs, generate responses in which the subthreshold response monotonically increases but remains very close to the baseline of the control situation. Incoherent feedforward loops may also produce hormetic responses if the compensation process, as represented by the feedforward signaling strength, is greater than that of perturbation. Many other network mechanisms, such as those in steroid hormone signaling, can also produce nonmonotonic responses to chemical toxicants (Conolly and Lutz 2004; Kohn and Melnick 2002; Li et al. 2007).

The tools described here for understanding network motifs and their dose–response properties should also be amenable to analysis of integrated networks in which groups of motifs work together in parallel or sequential patterns to control coordinated cellular responses. In these larger settings, the threshold response of a single protein or gene, especially if the protein is a transcription factor, can propagate to regulate coordinated responses that collectively control cellular phenotypes. Moreover, for motifs that do not have a perfectly flat subthreshold response, downstream motifs may filter out small changes in the subthreshold region, thereby producing distal cellular responses with clear thresholds.

Adverse outcome pathways (AOPs) describe the processes from molecular initiating events through toxicity pathways and cellular and organ responses, resulting in apical responses in exposed individuals (Ankley et al. 2010; Vinken 2013). Molecular network motifs operating in cells act at an immediate step in the AOP, propagating the perturbation associated with chemical exposure on to higher levels of organization (i.e., the cell, tissue, organ, and organism). These motifs sit at the toxicity pathway level of the AOP. When early key events (those that are necessary but not sufficient for the adverse outcome) arise through processes that overwhelm these network motifs, threshold responses of the motifs should also propagate into the larger AOP. Because of the multiplicity of events in any AOP, the threshold in the apical responses may actually occur at higher doses/perturbations than those associated with thresholds in the key network motif.

Stress pathway function appears to involve both rapid, posttranslational signaling for smaller, transient deviations from basal function and slower activation of transcriptional responses for more persistent, higher-level stresses (Mettetal et al. 2008). The loss of control corresponds to a tipping point and a change from an adaptive response to overt toxicity. More rigorous evaluations of stress pathways could redirect experimental studies from an insular focus on transcriptional programs (Simmons et al. 2009) and give more attention to the rapid posttranslational programs that likely maintain cellular homeostasis through integral feedback within the canonical stress pathway motifs (Muzzey et al. 2009). The combination of posttranslational and transcriptional arms of a coordinated signaling ensemble provides more flexibility in cellular response patterns for different intensities and duration of pathway perturbations. In addition to stress pathway activation, toxic responses associated with receptor-mediated pathways appear to involve activation of cellular programs controlling proliferation or differentiation. Ultrasensitive motifs involving feedback processes clearly play roles in these higher-level responses, coordinating contributions from suites of individual motifs (Csikasz-Nagy et al. 2006). For example, models of platelet-derived growth factor signaling through receptor tyrosine kinases and HU-1 signaling through GPC-receptor pathways coordinate MAPK cascades and integrated downstream cellular responses (Bhalla et al. 2002; Bromberg et al. 2008).

Putting knowledge of network motifs into practical use for low-dose extrapolations requires an understanding of the primary network structure of the toxicity pathway affected by the chemical stressor. Some quantitative measures of pathway biomarkers (e.g., genes, proteins) may be needed to determine the dominant interactions in specific feedback or feedforward motifs. With this information in hand, the structure of the primary network would then support inferences about expected dose–response behaviors, including thresholds. Additional experimental data might be needed to differentiate possibilities for specific variations of some of the motifs, for example, in distinguishing between proportional and integral feedback controls or among feedforward controls with different signaling strengths in the feedforward arm. Many cellular signaling pathways utilize combinations of motifs. For example, in stress-response pathways, rapid, robust adaptation often arises by coupling negative feedback and incoherent feedforward loops (El-Samad et al. 2005; Zhang et al. 2009). In these situations low-dose extrapolations need to consider the concerted action of the interconnected network motifs.

Although the threshold motifs presented here primarily function at the level of intracellular molecular networks, the overall concepts will also be applicable to higher-level biological hierarchies in which cells, tissues, and organs are the interacting components, which are connected by paracrine, endocrine, and neural signals into large-scale biological networks. A clear understanding of the network motif context in which biological responses are examined will help in predicting the shape of the responses in low-dose regions. Nevertheless, biological variability existing among individuals (e.g., individual cells, individual humans, other biological entities) complicates estimation of the shape of dose–response curves and the specific doses associated with thresholds. These variations will result in heterogeneous responses to identical chemical perturbations among individuals. In theory, if each individual has a non-zero, different threshold for a particular stressor, then the population-averaged response should exhibit a threshold comparable to the lowest individual threshold values. The robust nature of biological systems can ensure that healthy individuals respond in a qualitatively similar fashion despite large parameter variations in the underlying components (Kitano 2004). However, in a large population, there could be “abnormal” individuals who have no threshold or individuals who have thresholds below background exposure levels, in which case the population-averaged response would show no threshold. In addition, there may be cases where the perturbations produced by a chemical can add to an existing disease process. Such considerations have led to the suggestion that thresholds can only be defined for individuals (or individual cells) and that population heterogeneity will tend to linearize the averaged dose response and obscure the threshold (Lutz 1990, 2001; White et al. 2009; Zhang et al. 2010b). Despite such an argument, if there is clear mechanistic evidence that a normal individual does exhibit a threshold for a particular stressor, then it will be always reassuring in knowing that those who have thresholds above the exposed dose are fully protected. This is a completely different risk assessment scenario than the case of a universal nonthreshold model where every individual faces some increments in health risk at any exposure levels. A better approach in the future, as is slowly but steadily being done with personalized medicine—is to implement personalized risk assessment by stratifying the population according to their individual threshold levels. To this end, understanding the mechanisms of biological thresholds is necessary.

In the future, toxicity testing is likely to rely increasingly on *in vitro* test results for conducting risk and safety assessments (Andersen and Krewski 2010; NRC 2007). Interpretation of these tests will rely on understanding toxicity pathways and the expected shapes of dose–response curves arising for pathway perturbation by toxic chemicals (Rossini and Hartung 2012). Mechanistic cellular toxicity studies should include a greater consideration of the underlying network motifs regulating cellular-level responses. Understanding the quantitative aspects of network motifs relevant in toxicity pathway perturbations will be an integral component for these cell-based dose–response assessments and for training future toxicologists. The inclusion of more mechanistically oriented thinking into conventional cellular and molecular toxicology curricula is necessary for a better appreciation of cellular-level dose–response behaviors.

## Supplemental Material

(188 KB) PDF
