# Supplementary material for: Molecular Signaling Network Motifs Provide a Mechanistic Basis for Cellular Threshold Responses
Source: Environ Health Perspect. 2014 Aug 12;122(12):1261–70. doi: 10.1289/ehp.1408244 (PMC4256703; doi:10.1289/ehp.1408244)
Supplement: (188 KB) PDF [file ehp.1408244.s001.508.pdf]

Supplemental Material

Molecular Signaling Network Motifs Provide a Mechanistic Basis for Cellular Threshold Responses

Qiang Zhang, Sudin Bhattacharya, Rory B. Conolly, Harvey J. Clewell III, Norbert E. Kaminski, and Melvin E. Andersen

Table of Contents **Pages**

**Proportional feedback control**	2
**Integral feedback control**	3
**Incoherent feedforward control**	4
**Saddle-node bifurcation**	5
**Pitchfork bifurcation**	6
**Transcritical bifurcation**	7
**Molecular titration**	9
**References**	10

The following .xml files are provided in a separate file (ehp.1408244.s002.zip):

SBML_1_Proportional_Feedback_Control.xml

SBML_2_Integral_Feedback_Control.xml

SBML_3_Incoherent_Feedforward_Control.xml

SBML_4_Saddle_Node_Bifurcation_I.xml

SBML_4_Saddle_Node_Bifurcation_II.xml

SBML_5_Pitchfork_Bifurcation.xml

SBML_6_Transcritical_Bifurcation_I.xml

SBML_6_Transcritical_Bifurcation_II.xml

SBML_7_Molecular_Titration.xml

URL for all xml files: http://ehp.niehs.nih.gov/wp-content/uploads/advpub/2014/8/ehp.1408244.s002.zip

Proportional feedback control

The following ordinary differential equations (ODEs) describe proportional feedback control (Figure 2A in main text):

$$dY/dt = k_0 + k_1S - k_2GY, \quad (1)$$

$$dG/dt = k_3T^{n1}/(J_1^{n1} + T^{n1}) - k_4G, \quad (2)$$

$$dT/dt = k_5Y^{n2}/(J_2^{n2} + Y^{n2}) - k_6T. \quad (3)$$

For simplicity, we omitted posttranslational feedback (dashed line in Figure 2A in main text); however, its exclusion does not alter the basic conclusions. The Hill terms in Eqs. (2) and (3) describe ultrasensitive induction of G by T and ultrasensitive activation of T by Y, respectively. The steady-state value of T is related to the steady-state value of Y, and the steady-state value of G is in turn related to the steady-state value of T. This interrelationship ensures that the state of the controller reflects the current value of the controlled variable. After an initial perturbation, proportional feedback allows the controlled variable Y to dynamically adapt to the continued presence of stressor S (Figure 2B in main text, S=0.3, 0.6, 0.9, and 1.2 with increasingly darker red lines). The "behind-the-scene" process underlying adaptation is activation of T leading to induction of G. The ability of Y to return close to baseline depends on the degree of ultrasensitivity, or signal amplification, of the feedback loop, provided by the Hill coefficients n_1 and n_2 (Zhang and Andersen 2007). Proportional feedback control does not produce perfect adaptation (i.e., a true threshold) and the steady-state Y vs. S curve in the low-dose region is

monotonically increasing (Zhang and Andersen 2007). However, with sufficiently high amplification, the low-dose region becomes virtually indistinguishable from the baseline, thus giving a very low response in this portion of the curve (Figure 2C in main text). At high stressor levels where the induction of G becomes saturated, Y rises sharply from the baseline. Model parameters: k_0=10, k_1=10, k_2=10, k_3=0.2, k_4=0.1, k_5=500, k_6=0.5, J_1=1, J_2=2, n1=10, n2=10. SBML code for this model is provided in a separate Supplemental Material file (see SBML_1_Proportional_Feedback_Control.xml).

Integral feedback control

The following ODEs describe integral feedback control (Figure 2D in main text):

$$dY/dt = k_0 + k_1S - k_2GY, \tag{4}$$

$$dG/dt = k_3T/(J+ T) - k_4G, \tag{5}$$

$$dT/dt = k_5Y - k_6. \tag{6}$$

As for proportional feedback control, we simplify the model code by excluding any posttranslational regulation. The degradation step for T in Eq (6) is a zero-order process (represented by the -k_6 term), which provides the integrator function. T would reach a steady state only when Y settles to a preset value $Y_0=k_6/k_5$ (baseline level). When Y rises above the baseline level (Y_0) due to perturbation by S, the production rate of T (k_5Y) exceeds the degradation rate (k_6). Thus, T increases, and the amount of newly synthesized T becomes the time integral of the difference between the production rate and degradation rate. Increased T induces G to a higher level to reduce Y. As long as Y is still above the baseline, the production rate of T continues to exceed its degradation rate. As a result, T continues to increase leading to

further induction of G, until Y returns to the baseline Y_0 (Figure 2E in main text), where the system reaches a steady state. This way integral control produces perfect adaptation. The stressor level at which the integral control fails, as the induction of G reaches a maximum, defines the threshold of S. At stressor levels below the threshold, the controlled variable settles to a steady-state level identical to the non-stressed baseline level (Figure 2F in main text). Model parameters: k_0=10, k_1=10, k_2=10, k_3=0.2, k_4=0.1, k_5=0.5, k_6=0.5, J=1. SBML code for this model is provided in a separate Supplemental Material file (see SBML_2_Integral_Feedback_Control.xml).

Incoherent feedforward control

The following ODEs describe incoherent feedforward control (Figure 3A, 3D and 3G in main text):

$$dY/dt = k_0 + k_1S - k_2GY, \tag{7}$$

$$dG/dt = k_3 + k_4T - k_5G, \tag{8}$$

$$dT/dt = k_6S(T_{tot} - T) - (k_7(T_{tot} - T) + k_8)T. \tag{9}$$

In contrast to the feedback motifs where Y regulates T, here the stressor S directly regulates T. An autocatalytic covalent modification process driven by S activates T. The magnitude of the feedforward signaling strength (referred to as gain) quantifies the induction of G by S (via T). Depending on the value of the feedforward gain compared to the perturbation gain (change in Y directly caused by S), there are three different scenarios. When the feedforward gain is smaller than the perturbation gain (Figure 3A in main text), adaptation is partial (Figure 3B in main text) and the steady-state dose-response curve is close to but not identical to the baseline in the low-dose region (Figure 3C in main text). When the feedforward gain matches the perturbation gain

(Figure 3D in main text), there is perfect adaptation (Figure 3E in main text) leading to a threshold (Figure 3F in main text). The threshold occurs when T or G reach their limit of activation. When the feedforward gain exceeds the perturbation gain (Figure 3G in main text), the induced stress-gene activity overcompensates for the change of Y by S. As a result, there is over-adaptation (Figure 3H in main text), resulting in a hormetic, J-shaped dose response (Figure 3I in main text) (Kaplan et al. 2008; Kim et al. 2008). In each of the three cases, the value of k_4 was varied (k_4 =0.09, 0.1, and 0.11 respectively) to alter the feedforward gain. Model parameters: k_0=10, k_1=10, k_2=10, k_3=0.1, k_4=0.1, k_5=0.1, k_6=1, k_7=1, k_8=0.0001, T_{tot}=1. SBML code for this model is provided in a separate Supplemental Material file (see SBML_3_Incoherent_Feedforward_Control.xml).

Saddle-node bifurcation

The following ODEs describe positive feedback with saddle-node bifurcation (Figure 4A in main text):

$$dG_1/dt = k_1G_2S - k_2G_1, \quad (10)$$

$$dG_2/dt = k_3 + k_4G_1^n/(J_1^n + G_1^n) - k_5G_2. \quad (11)$$

Here G_1 and G_2 are mutually-activating genes forming a positive feedback loop. S is an external signal stimulating G_1. The Hill function in Eq. (11) introduces ultrasensitivity, a necessary nonlinear term for generating bistability (Angeli et al. 2004; Zhang et al. 2013). Depending on the level of S, both G_2 (Figure 4B in main text) and G_1 (not shown) settle into one of two discrete states. The steady-state dose response behavior between S and G_2 displays a saddle-node bifurcation (Figure 4C in main text). At low levels of S, G_2 increases slightly as S increases.

When a threshold value of S ("on-threshold") is exceeded, the positive feedback pushes the system to the second stable steady state with G_2 and G_1 switching abruptly to higher levels. Once in the on-state, the bistable system does not switch off immediately to the off-state even when S is reduced. The system switches off only when S decreases to a further lower level ("off-threshold"). Between the off- and on-threshold values of S, the system can be either on or off (thus the term "bistable"), with unstable saddle-node points (blue dashed line in Figure 4C in main text) lying in between. Model parameters: k_1=0.392, k_2=1, k_3=1, k_4=9, k_5=1, J_1=1, n=5. SBML code for this model is provided in a separate Supplemental Material file (see SBML_4_Saddle_Node_Bifurcation_I.xml).

An alternative network motif structure to generate saddle-node bifurcations is a double-negative feedback loop (Figure 4D in main text), described with the following ODEs:

$$dG_1/dt = k_1J_1/(J_1 + G_2) - k_2G_1 - k_3SG_1, \qquad (12)$$

$$dG_2/dt = k_4 + k_5J_2^n/(J_2^n + G_1^n) - k_6G_2. \qquad (13)$$

The dynamic and bifurcation behaviors of the system are similar to the positive feedback example except that G_1 changes in an opposite direction compared to G_2 (Figure 4E and 4F in main text). Model parameters: k_1=40, k_2=1, k_3=1.72, k_4=1, k_5=9, k_6=1, J_1=0.2, J_2=1, n=5. SBML code for this model is provided in a separate Supplemental Material file (see SBML_4_Saddle_Node_Bifurcation_II.xml).

Pitchfork bifurcation

The following ODEs describe a supercritical pitchfork bifurcation motif (Figure 5A in main text):

$$dG_1/dt = k_1 + (k_2S + k_3)J^n/(J^n + G_2^n) - k_4G_1, \quad (14)$$

$$dG_2/dt = k_1 + (k_2S + k_3)J^n/(J^n + G_1^n) - k_4G_2. \quad (15)$$

The bifurcation diagrams show the behavior of gene G_1 (Figure 5C in main text) and gene G_2 (Figure 5E in main text) with respect to signal S. In the absence of S, G_1 and G_2 have equivalent low levels of expression because of the symmetry in the system. Since S has the same effect on both of them, G_1 and G_2 increase gradually with increasing S but remain equal. As S increases further, the system bifurcates, where any slight asymmetry between the two genes leads to activation of one gene and repression of the other due to mutual-inhibition. This stage is characterized by bistability: the existence of two mutually-exclusive stable steady states (i.e., high G_1 / low G_2 or low G_1 / high G_2). Unlike the abrupt saddle-node bifurcation, here the transition from monostability to bistability occurs gradually through the *supercritical* pitchfork bifurcation (Strogatz 1994). The dynamics of the pitchfork bifurcation system in response to various levels of S is shown in Figure 5B and 5D in main text. For these simulations, the system starts with a small asymmetry: the initial value of G_1 is slightly higher than G_2. For subthreshold S levels, both G_1 and G_2 rise gradually. Once S exceeds the pitchfork bifurcation threshold, the activation of G_1 occurs first, leading to downregulation of G_2. Model parameters: k_1=0.75, k_2=0.77, k_3=0.5, k_4=1, J=1, n=4. SBML code for this model is provided in a separate Supplemental Material file (see SBML_5_Pitchfork_Bifurcation.xml).

Transcritical bifurcation

The following ODEs describe two different transcritical bifurcations with thresholds. The first gives behavior equivalent to that in Figure 6A in main text:

$$dR/dt = k_1SR - k_2R - k_3R^2. \quad (16)$$

Here gene R positively autoregulates its own expression and requires both R and the external signal, S, for induction. Two terms describe degradation of R - a linear term k_2R and a second term k_3R^2 that represents enhanced degradation as R increases. This system displays two steady states, a horizontal line depicted by $R_{ss}=0$ and a straight, slanted line depicted by $R_{ss}=(k_1S-k_2)/k_3$. The bifurcation diagram in Figure 6C in main text shows the stability of the two steady states. At low levels of S, $R_{ss}=0$ is the stable steady state. Simultaneously, there is an imaginary unstable steady state with negative values of R. As S increases beyond the threshold, the two steady states exchange stability. The steady state represented by the horizontal line becomes unstable, while the one represented by the slanted line becomes the stable steady state. Since real-world systems only settle to stable steady states, the dose response of this transcritical bifurcation motif (Figure 6A in main text) follows the solid red line in Figure 6C in main text, which has a threshold. Model parameters: $k_1=1$, $k_2=1$, $k_3=1.2$. SBML code for this model is provided in a separate Supplemental Material file (see SBML_6_Transcritical_Bifurcation_I.xml).

The ODE below describes a transcritical bifurcation motif as in Figure 6D in main text:

$$dR^*/dt = k_0R^*R + k_1SR^*R - k_2R^*. \quad (17)$$

This ODE describes reversible modifications of protein substrate R to R^* in an autocatalytic manner. The total amount of R and R^* remains constant at $R_{tot} = R + R^*$. A transcritical bifurcation results as external signal S increases (Figure 6F in main text). As long as S is below the threshold, R^* remains at zero. As S crosses the threshold, R^* increases in a nonlinear fashion.

Model parameters: k_0=0.5, k_1=0.5, k_2=4. SBML code for this model is provided in a separate Supplemental Material file (see SBML_6_Transcritical_Bifurcation_II.xml).

Molecular titration

The following ODEs describe a molecular titration motif (Figure 7A in main text):

$$d[SR]/dt = k_1 S \cdot R - k_2[SR], \quad (18)$$

$$dG/dt = k_3 + k_4 S - k_5 G. \quad (19)$$

External signal S induces gene G. In addition, a high-affinity inhibitor R titrates S away into an inactive complex [SR]. In the simulation, the total amount of free R plus R bound in the [SR] complex is constant (R_{tot} = R + [SR]). The total amount of free S plus S bound in the complex [SR], i.e., S_{tot} = S + [SR], is varied as the input dose. Although this is not a threshold response, there is an input dose that causes a steep change in behavior with this motif (Figure 7C in main text). At low levels of S_{tot}, R sequesters most of the S molecules due to the high-affinity binding between S and R. There is little S available for inducing G. As the level of S_{tot} approaches R_{tot}, all R is bound by S. Any further increase in S_{tot} increases free S dramatically leading to induction of G above the baseline. Model parameters: k_1=100, k_2=1, k_3=1, k_4=1, k_5=1. SBML code for this model is provided in a separate Supplemental Material file (see SBML_7_Molecular_Titration.xml).

References

Angeli D, Ferrell JE, Jr., Sontag ED. 2004. Detection of multistability, bifurcations, and hysteresis in a large class of biological positive-feedback systems. Proc Natl Acad Sci U S A 101(7): 1822-1827.

Kaplan S, Bren A, Dekel E, Alon U. 2008. The incoherent feed-forward loop can generate non-monotonic input functions for genes. Mol Syst Biol 4: 203.

Kim D, Kwon YK, Cho KH. 2008. The biphasic behavior of incoherent feed-forward loops in biomolecular regulatory networks. Bioessays 30(11-12): 1204-1211.

Strogatz SH. 1994. Nonliear dynamics and chaos: with applications to physics, biology, chemistry, and engineering. 1st ed. Cambridge: Perseus Books Group.

Zhang Q, Andersen ME. 2007. Dose response relationship in anti-stress gene regulatory networks. PLoS Comput Biol 3(3): e24.

Zhang Q, Bhattacharya S, Andersen ME. 2013. Ultrasensitive response motifs: basic amplifiers in molecular signalling networks. Open Biol 3(4): 130031.
